# AIEgen-Peptide Bioprobes for the Imaging of Organelles

**DOI:** 10.3390/bios12080667

**Published:** 2022-08-22

**Authors:** Bochao Chen, Haotong Yuan, Wei Zhang, Jingjing Hu, Xiaoding Lou, Fan Xia

**Affiliations:** 1 State Key Laboratory of Biogeology and Environmental Geology, Faculty of Materials Science and Chemistry, China University of Geosciences, Wuhan 430078, China; 2 Department of Chemical and Environmental Engineering, University of Nottingham Ningbo China, Ningbo 315100, China

**Keywords:** organelles, peptide, fluorescence imaging, aggregation-induced emission

## Abstract

Organelles are important subsystems of cells. The damage and inactivation of organelles are closely related to the occurrence of diseases. Organelles’ functional activity can be observed by fluorescence molecular tools. Nowadays, a series of aggregation-induced emission (AIE) bioprobes with organelles-targeting ability have emerged, showing great potential in visualizing the interactions between probes and different organelles. Among them, AIE luminogen (AIEgen)-based peptide bioprobes have attracted more and more attention from researchers due to their good biocompatibility and photostability and abundant diversity. In this review, we summarize the progress of AIEgen-peptide bioprobes in targeting organelles, including the cell membrane, nucleus, mitochondria, lysosomes and endoplasmic reticulum, in recent years. The structural characteristics and biological applications of these bioprobes are discussed, and the development prospect of this field is forecasted. It is hoped that this review will provide guidance for the development of AIEgen-peptide bioprobes at the organelles level and provide a reference for related biomedical research.

## 1. Introduction

One of the reasons why eukaryotes are more complicated than prokaryotes is that eukaryotes contain many independent inner membrane systems, which are called organelles or subcellular compartments [1,2]. Each organelle has unique structural characteristics and plays a different role in various physiological processes. They maintain cell biochemical reactions efficiently, diversely and stably. The organelles of eukaryotes contain a cell membrane, mitochondria, a nucleus, lysosomes, an endoplasmic reticulum, a Golgi apparatus, lipid droplets, ribosomes, etc. [3]. These organelles can not only work independently but also cooperate with each other. On one hand, working independently could guarantee orderly physiological activities. For instance, mitochondria are mostly responsible for producing energy, controlling the apoptosis progress and regulating intracellular calcium and reactive oxygen species (ROS) [4,5,6]. Lysosomes contain many hydrolases that can hydrolyze multiple waste or abnormal proteins [7,8]. On the other hand, complicated physiological activities require the cooperation of multiple organelles, such as the synthesis of proteins. At first, proteins need to be transcribed in the nucleus and translated in ribosomes. Subsequently, they are modified and packaged by the Golgi apparatus to obtain the active proteins with intended functions. In this case, dysfunction of organelles was able to cause various diseases such as cancer, metabolic disorders, cardiovascular and neurodegenerative diseases and so on [9,10,11,12,13,14]. For example, when mitochondria are damaged, cytochrome c and mitochondria outer membrane proteins are released into the cytoplasm and interact with apoptotic protease activator 1, which leads to the activation and recruitment of the caspase family, ultimately resulting in the apoptosis of cells [15,16,17]. Thus, it is very important to image and detect these organelles. It helps us to study the different physiological and biochemical processes of cells, understand the operation mechanism of the life system and establish the foundation for biomedical research. 

Fluorescence imaging technology is widely used in various fields due to its non-invasiveness, high sensitivity and high temporal spatial resolution [18,19,20]. Aggregation-induced emission (AIE) is a phenomenon by which luminogen does not emit fluorescence in a dilute solution but emits strong fluorescence in a high concentration or solid state [21,22,23,24]. AIE luminogens (AIEgens) generally have a molecular rotor structure. In the free state, the molecular rotor can rotate freely and activate non-radiative transitions to consume excitation energy. In the aggregated state, molecular rotor movement is restricted, and excitation energy is mainly released through radiative transitions, which results in the bright and stable fluorescence emission [25,26,27]. In recent years, due to their outstanding properties, such as a high quantum yield, a resistance to photobleaching, a large Stokes shift and photosensitivity, AIEgens have been widely used in biomarker detection and imaging [28,29], drug delivery [30,31], surgery navigation [32,33], anti-bacteria processes [34], phototherapy [35,36,37,38,39,40] and a series of biomedical application areas. 

AIEgens can be conjugated with biological macromolecules (peptides, polysaccharides, nucleic acids, proteins, etc.) through covalent bonds [22,41,42]. Among them, peptides are widely developed in the biomedical field. When peptides bind to AIEgens, the addition of peptides restricts the rotation of AIEgens, which leads to AIEgens emitting fluorescence [27]. AIEgens have the advantages of photobleaching resistance, photosensitivity and a high quantum yield. At the same time, AIEgens are generally lipid-soluble and more hydrophobic and enter the cell difficultly. The introduction of peptides can improve the hydrophilicity of AIEgens and make them have good biocompatibility, which is conducive to the application of bioprobes in the biomedical field. In addition, the introduction of peptides can also give AIEgens various functions, such as the specific recognition function, toxic function, targeting function, etc. Simultaneously, the immunogenicity of bioprobes can be reduced. Therefore, the design of biomacromolecule-functionalized AIEgens can be used as a practical tool to obtain complex information at the biological level, analyze biomacromolecule interactions and understand disease mechanisms. At the same time, peptides are unstable and easily degraded by bioactive substances. We hope that obstacles can be overcome in future research [43,44,45,46]. 

**Figure 1:**
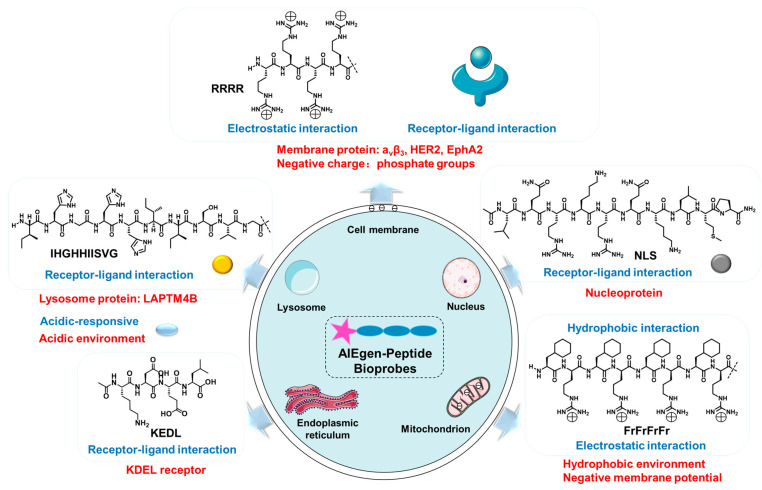
The mechanism of bioprobes-targeting organelles.

There are various strategies for designing peptide-targeting organelles. Among them, the strategy for the design of cell membrane-targeted peptides is the electrostatic interaction of arginine rich in positive charge with phosphate groups on the cell membrane. In addition, the cell membrane can be targeted by peptides that can bind to cell membrane receptors. The nuclear localization peptide (NLS) peptides could bind to the nucleoprotein. The peptides targeting the mitochondria are usually liposoluble cationic peptides because mitochondria have negative membrane potential and a hydrophobic environment. The lysosomal-targeted peptides are generally reactive under acidic conditions and can bind to lysosomal proteins. ER-targeted peptides could bind to the KDEL receptor (Figure 1). According to different organelles, this article reviews the recent progress of AIEgen-peptide bioprobes in organelles imaging. The structural characteristics and application strategies of AIEgen-peptide bioprobes in different organelles are summarized. We mainly study organelles containing cell membranes, mitochondria, nuclei, lysosomes and endoplasmic reticula (Table 1). We hope this review will provide guidance for the development of AIEgen-peptide bioprobes at the organelles level and provide a reference for related biomedical research. 

## 2. Design Strategies and Recent Examples

In this section, we summarized AIEgen-peptide bioprobes according to the organelle types. Based on the characteristics of organelles, the key design strategies and the targeting mechanism of bioprobes were analyzed. Additionally, their biological applications were described for each organelle.

### 2.1. Cell Membrane-Targeted Bioprobes

**Figure 2:**
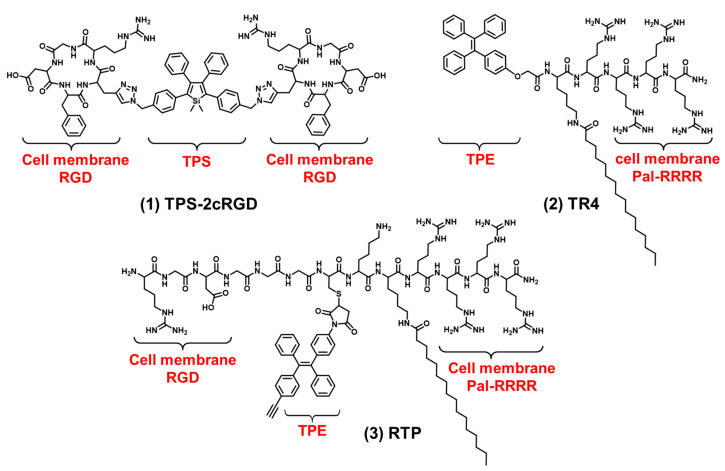
The chemical structures of cell membrane-targeting bioprobes.

The cell membrane is composed of an amphiphilic phospholipid bilayer with a large negative potential. It plays an important role in maintaining homeostasis, controlling substance transport and regulating signal transduction [16,17,47]. At present, cell membrane-targeting fluorescent probes usually consist of a fluorophore and an anchoring element [15]. The cell membrane-anchoring element is generally the substance including: penetrating peptides [48], alkyl chains [49], cholesterol [50], protein ligands [51], antibodies, aptamers [52], etc. Protein ligands can be utilized to target cell membrane receptors; for example, a peptide sequence RGD often acts as a targeted peptide of the integrin receptor in the cell membrane. As shown in Figure 2 compound (**1**), Liu et al. reported a new bioprobe TPS-2cRGD by integrating an AIEgen (TPS) with cyclic arginine-glycine-aspartic tripeptide (cRGD), a targeting ligand to the cell membrane integrin α_v_β_3_ receptor [53]. The excitation wavelength of the bioprobe is 356 nm, and the maximum emission wavelength is 480 nm. The bioprobe overlapped well with commercial cell membrane dyes and was used to track α_v_β_3_-positive cancer cells. The negative membrane potential and lipid solubility of the phospholipid bilayer lead to the enrichment of lipophilic and cations molecules in the cell membrane. Because of the abundant positive charge, cell-penetrating peptide RRRR was usually used to improve the targeting ability of the cell membrane. At the same time, palmitic acid was usually used to target the cell membrane due to its lipid solubility. Liang et al. reported an AIEgen-peptide bioprobe TR4 [54]. As shown in Figure 2 compound (**2**), the bioprobe consisted of three elements: palmitic acid, cell-penetrating peptide (RRRR) and tetraphenylethylene (TPE). The excitation wavelength of the bioprobe is 330 nm, and the maximum emission wavelength is 466 nm. When MCF-7 cells were incubated with TR4, the cell membrane was labeled. TR4 showed good photostability and biocompatibility and low toxicity. It opened the door of the TR4 in cell membrane imaging. To further improve cell membrane-targeting ability, our group combined RRRR, RGD and palmitic acid to AIEgen to track the imaging of the cell membrane [55]. As shown in Figure 2 compound (**3**), RTP (λ_ex_: 330 nm, λ_em_: 500 nm) consisted of three elements: a RGD-targeted peptide, a palmitic acid-modified hydrophilic peptide (Pal-RRRR) and AIEgen (T-MY). The palmitic acid had a cell membrane-targeting function and was inserted into the cell membrane through hydrophobic interactions. A RGD-targeted peptide was bound to integrin receptors on the cell membrane. A hydrophilic peptide (RRRR) was bound to the cell membrane through electrostatic interactions. Under the comprehensive combination of RGD and Pal-RRRR, RTP successfully achieved the imaging of the cell membrane precisely and robustly. Simultaneously, RTP showed durable stability and a strong resistance to photobleaching.

**Figure 3:**
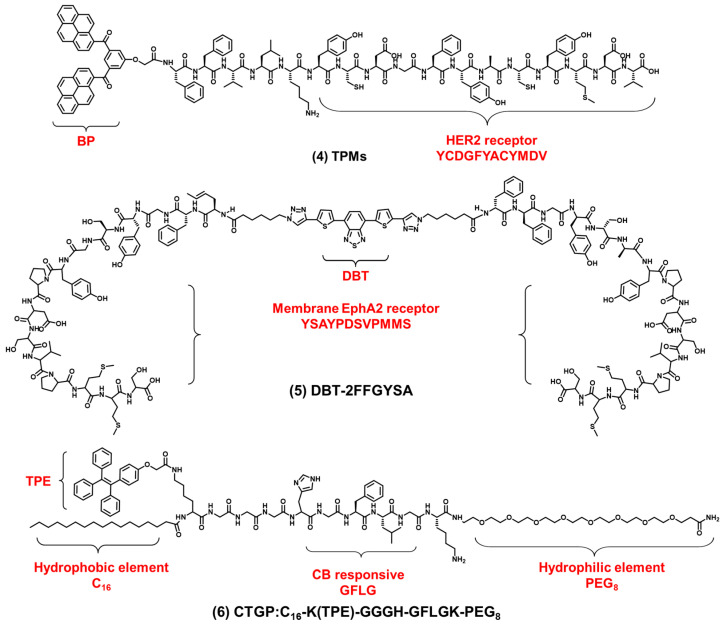
The chemical structures of cell membrane-targeting bioprobes relying on the cell membrane protein.

In addition to the integrin receptors overexpressed in most cancer cells, some cancer cells also have specific receptors. For example, human epidermal growth factor receptor 2 (HER2) is overexpressed in breast cancers. HER2 is a tyrosine receptor kinase that can induce dimer formation with itself and then lead to downstream signal activation. Wang et al. designed a AIEgen-peptide bioprobe (TPM) that targeted the HER2 receptor on the cell membrane [56]. As shown in Figure 3 compound (**4**), the bis-pyrene (BP) element with the AIE property for fluorescence reporting, the peptide (YCDGFYACYMDV) bound to the HER2 receptor on the surface of cell membrane and the peptide (KLVFF) was used as a hydrophobic element that could be assembled into nanofibers. When the peptide bound to HER2 on the cancer cells’ surface, TPM (λ_ex_: 380 nm, λ_em_: 520 nm), was converted into nanofibers and attached to the cell membrane strongly, this restricted the rotation of AIEgen and the imaged cell membrane. In addition, TPM also destroyed the formation of HER2 dimers, thereby blocking the downstream signaling pathway and leading to tumor cell apoptosis. This is a bioprobe targeting cell surface receptors in order to target the cell membrane which has favorable potential for future clinical applications. In addition, Eph receptor A2 (EphA2) is an adrenaline tyrosine receptor kinase overexpressed in tumor-specific membranes. EphA2 plays an important role in promoting cancer malignancy. Therefore, the specific imaging of EphA2 is of great significance for the diagnosis of tumors. Ding et al. constructed a self-assembling bioprobe (DBT-2FFGYSA) that selectively targeted the EphA2 protein on the cell membrane [57]. As shown in Figure 3 compound (**5**), it consisted of three elements: the middle element was AIEgen (DBT), the two aromatic phenylalanine (FF) were the self-assembled element and the peptide sequence YSAYPDSVPMMS (YSA) could specifically bind to EphA2. The excitation wavelength of the bioprobe is 490 nm, and the maximum emission wavelength is 642 nm. The bioprobe selectively targeted EphA2 receptors and caused AIEgen to aggregate in the cell membrane. In addition, this bioprobe could effectively transform cold tumors into hot tumors to stimulate immune response and inhibit tumor growth. In addition to the strategy of the bioprobe bound to the cell membrane receptors, 16-carbon alkyl chains can adhere firmly to the cell membrane. Zhang et al. reported a membrane-targeted AIEgen-peptide bioprobe (CTGP) imaging the cell membrane [58]. As shown in Figure 3 compound (**6**), it was composed of the AIEgen element (TPE), cathepsin B (CB) enzyme-responded peptide element (GFLG) and 16-carbon alkyl chain element (C_16_), which targeted cell membrane. The excitation wavelength of the bioprobe is 370 nm, and the maximum emission wavelength is 470 nm. The bioprobe was cleaved by CB when the bioprobe was present in tumor cells with CB overexpression. CTGP was transformed from spherical nanoparticles into nanofibers. TPE was encapsulated by nanofibers on the cell membrane, so CTGP can image the cell membrane obviously. In addition, this encapsulation characteristic could prevent the DOX efflux in tumor cells and prevent the drug resistance of tumor cells from inhibiting the drug efflux. This encapsulation of the cell membrane opened a new avenue for tumor imaging and drug resistance research.

### 2.2. Nucleus-Targeted Bioprobes

The nucleus is the regulatory center of the genetic metabolism and the main place for the storage, replication and transcription of genetic information in cells. Peptides that target the nucleus have been widely developed in recent years. Conventional nuclear-targeted peptides include: the NLS peptide, the RrRK peptide, etc. [59]. One of the most frequently used peptides is NLS. The NLS peptide, a short chain of basic amino acids derived from the SV-40 virus, has been widely used in nuclear cargo delivery. Most fluorescence probes target the nucleus by electrostatic interaction with DNA or RNA. The NLS peptide targets the nucleus by binding to importin-A, which in turn binds to importin-B to form complexes and enters the nucleus directly through the nuclear pore [60,61,62,63,64]. So, the NLS peptide is widely used in nuclear imaging. 

**Figure 4:**
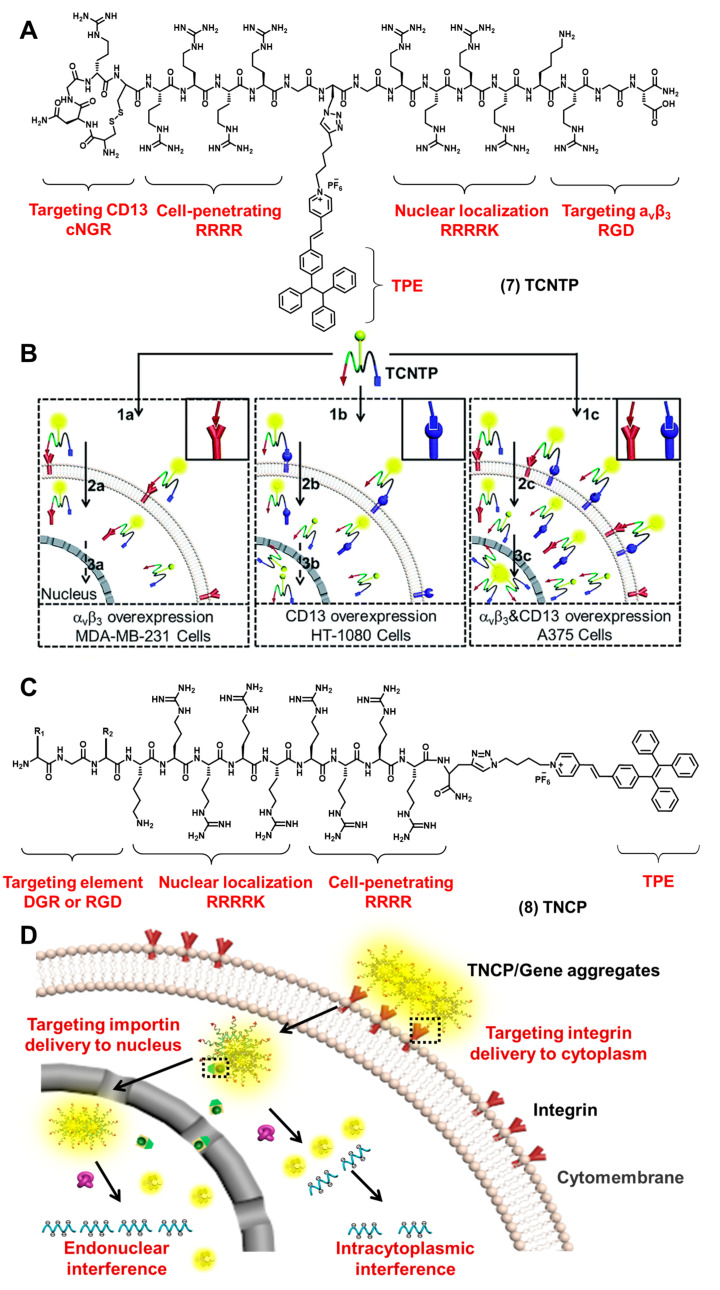
(**A**) The chemical structures of TCNTP. (**B**) The schematic illustration of TCNTP entering nucleus step by step by binding to the α_V_β_3_ and CD13 receptors (permission to reprint this figure has been requested from [65]). (**C**) The chemical structures of TNCP. (**D**) The schematic illustration of TCNP/ASO aggregates for stepwise delivery and regional therapeutics (this figure was adapted from [66] with some modifications).

To image the nucleus efficiently and specifically, our group reported a multifunctional bioprobe (TCNTP) that combined NLS peptides with AIEgen [65]. As shown in Figure 4A compound (**7**), TCNTP consisted of four elements: a targeted peptide (cNGR or RGD), a cell-penetrating peptide (CPP), NLS and an AIEgen element (PyTPE). The excitation wavelength of the bioprobe is 405 nm, and the maximum emission wavelength is 570 nm. TCNTP specifically bound to aminopeptidase N (CD13) and integrin α_v_β_3_ through cNGR or RGD under the action of the cell-penetrating peptide (Figure 4B). TCNTP entered into the cytoplasm efficiently and was transported into the nucleus with the help of NLS. Compared with the traditional commercial nuclear imaging dye Hoechst 33,258, TCNTP had a harmless physical internalization process and a durable photostability, which ensured its potential application in long-term tumor cell tracking.

After we achieved the imaging and tracking of the nucleus specifically, our group further proposed a gene delivery strategy to deliver antisense oligonucleotides (ASO) to the nucleus effectively. As shown in Figure 4C compound (**8**), our group designed and synthesized TNCP/ASO aggregates (λ_ex_: 405 nm, λ_em_: 580 nm) [66]. By changing the RGD-targeted element, a pair of molecules was synthesized (TDNCP and TRNCP). They were mainly composed of cell membrane-targeted peptides (RGD or DGR), cell-penetrating peptides (RRRR), nuclear targeted peptides (RRRRK) and the AIEgen element (PyTPE). It had favorable nuclear imaging ability and the ability to deliver therapeutic genes (Figure 4D). TNCP specifically bound to integrin receptor a_v_β_3_ and internalized into the cytoplasm. The positive charge of the cell-penetrating peptide (RRRR) could bind to therapeutic genes through electrostatic interaction, thereby shielding its positive charge and reducing cation toxicity. The nuclear localization element (RRRRK) could combine with the importin protein to enter the nucleus. All of the results demonstrated that TNCP enabled the sequential targeting, real-time tracking and efficient encapsulation of the therapeutic genes in the nucleus. At the same time, Liu et al. also designed a bioprobe using the NLS nuclear-targeted peptide (TPE-NLS) [67]; the bioprobe had good water-soluble and nuclear permeability. The bioprobe could be used to image the nucleus well, and it could overlap well with the commercial nuclear dye DRAQ5. Nucleus proteins were also used as a targeted substance. Jiang et al. reported a probe L2P4 that can target nuclear EB nuclear antigen 1 (EBNA1) [68]. L2P4 is composed of three parts: a viscosity-sensitive fluorescent molecule, the nuclear-targeted peptide (RrRK) and the EBNA1 inhibitory peptide (YFMVF). L2P4 played a major role in imaging EBNA1 in the nucleus. They developed the application potential of EBNA1 as a therapeutic target protein.

### 2.3. Mitochondria-Targeted Bioprobes 

Mitochondria are the energy factories with a double membrane. They perform aerobic respiration to produce a large amount of adenosine-triphosphate (ATP). They are the principal spots of the tricarboxylic acid cycle and oxidative phosphorylation. Mitochondria can generate ROS, such as H_2_O_2_ and HClO. The imbalance of ROS may result in extreme situations such as oxidative and reductive stresses, with the consequent onset of cell death [69]. Many diseases are linked to mitochondrial dysfunction, such as cancer, neurodegeneration and metabolic disorders. It is of great significance to develop mitochondrial fluorescent probes to monitor the morphology and function of mitochondria.

Mitochondria have a hydrophobic and dense double-membrane system whose membrane potential is −180 mV. Lipophilic and positively charged molecules can accumulate in mitochondria [70]. Fluorescent probes targeting mitochondria generally include the following structures: triphenyl-phosphonium (TPP), quaternary ammonium salt, pyridine and quinoline derivatives, cyanine derivatives and rhodamine derivatives [5,71,72]. Kwak et al. used triphenylphosphorus to target mitochondria, and a phenylalanine dipeptide (FF) achieved mitochondria-localized self-assembly to form nanofibers and achieved the imaging of mitochondria [43]. The peptide sequences that can target mitochondria include: Szeto–Schiller (SS) peptides (DMTrFK) [73], mitochondria-penetrating peptides (MPP, FrFKFrFK) [70,74,75], mitochondria-toxic peptides (KLAKLAKKLAKLAK) [6], etc. TPP is composed of a hydrophilic positive charge element and a hydrophobic element. Therefore, driven by the negative mitochondrial membrane potential and the lipophilic environment, TPP can be carried to target the inner mitochondria very quickly without any barrier. It is a delocalized lipophilic cation (DLC). When a large number of TPP accumulates in the mitochondria, its positive charge may lead to a loss of membrane potential and affect the electron transport in the respiratory chain, which easily causes higher cytotoxicity. Mitochondrial permeation peptides (MPPs) composed of alternating arrangements of cations and lipophilic residues can avoid the problem of the large aggregation of cations, and these peptides have a higher mitochondrial localization ability. 

**Figure 5:**
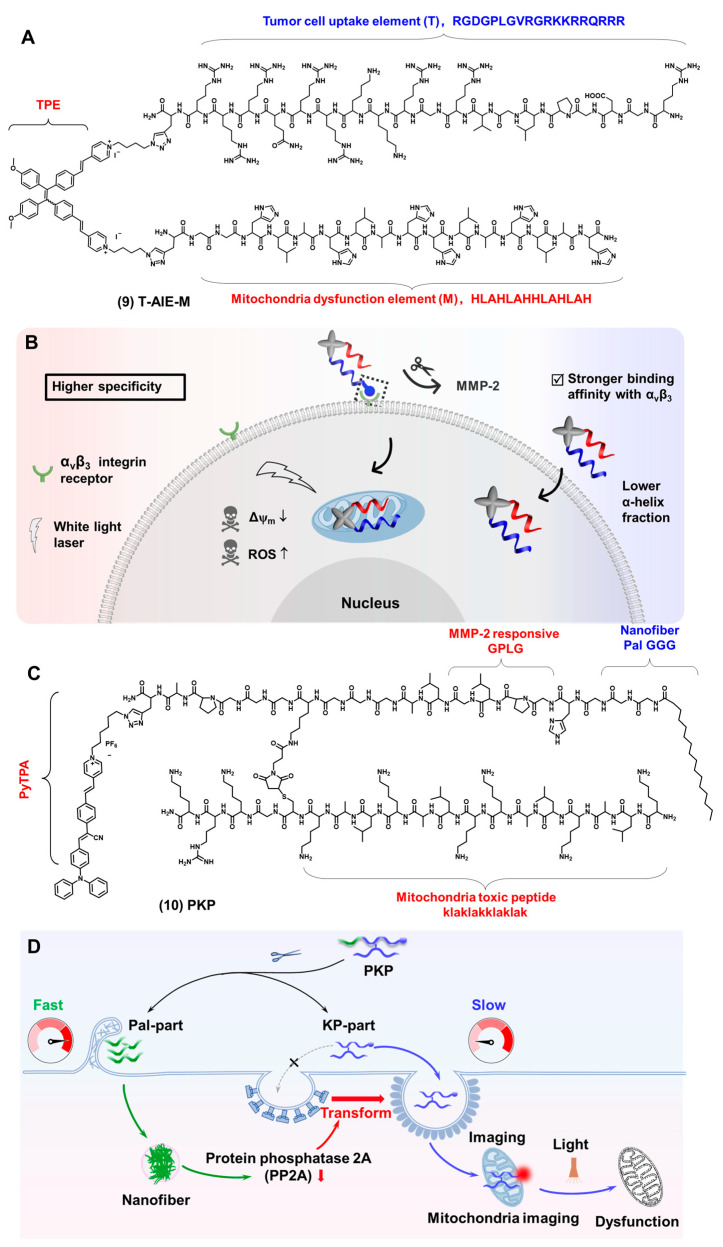
(**A**) The chemical structures of T-AIE-M. (**B**) The schematic illustration of the two probes with different fluorescence imaging and tumor inhibition properties (this figure was adapted from [76] with some modifications). (**C**) The chemical structures of PKP. (**D**) The schematic illustration of PKP entering the cell, leading to mitochondrial damage (this figure was adapted from [77]).

As shown in Figure 5A compound (**9**), our group reported a series of AIEgen-peptide bioprobes to target mitochondria. TPE derivatives were used as small molecule scaffolds [76]. The tumor cell uptake element (Element T) was used to enhance tumor cell internalization, whose sequence is RGDGPLGVRGRKKRRQRRR. The mitochondria dysfunction element (Element M) was used to induce mitochondrial dysfunction, whose sequence is HLAHLAHHLAHLAH. The excitation wavelength of the bioprobe is 420 nm, and the maximum emission wavelength is 720 nm. The mitochondrial co-localization coefficients of all these bioprobes were all above 0.7, which had a beneficial effect on mitochondrial imaging. By changing the spatial arrangement of different elements in the AIEgen element, we studied the effect of different spatial arrangements on the performance of these probes. All of the results showed that T-AIE-M have a higher specificity for tumor cells, while TM-AIE showed a stronger toxicity (Figure 5B). This work elucidated the effects of different element performances, opening the door for bioprobes-designed strategies and functionalization studies. To avoid lysosomes degradation and increase mitochondrial delivery efficiency, our group reported an AIEgen-peptide bioprobe PKP (Figure 5C compound **10**) that was divided into the Pal-part and KP-part [77]. The Pal-part entered cells and formed nanofibers to cause the decrease in the protein phosphatase (PP2A), which led to the KP-part entering the cell through caveolae-mediated endocytosis (CvME) and reaching the mitochondria, finally achieving mitochondria imaging and damage (Figure 5D). The mitochondrial delivery efficiency of the KP-part greatly improved the efficiency of the image-guided therapy. In addition, the bioprobe also showed accurate image-guided cancer therapy, which was expected to significantly expand its application and promote the development of personalized imaging and therapy.

### 2.4. Lysosome-Targeted Bioprobes

Lysosomes have a single layer membrane, a vesicle-like structure of 0.025–0.8 μM, an acidic environment and a pH value of 4.5–5.0 and contain a variety of hydrolytic enzymes that can hydrolyze discarded or abnormal proteins, nucleic acids, polysaccharides, etc. [78]. As cells age, they release hydrolases that digest and kill cells. Lysosomes play an important role in physiological processes such as metabolism and apoptosis. The mechanisms for targeting lysosomes can be roughly divided into two categories: (1) based on the acidic environment within lysosomes, (2) based on specific membrane proteins and hydrolases within lysosomes. 

Cathepsin B (CB) is overexpressed in cancer cell lysosomes, and the peptide GFLG is typically used as the specific response of the CB enzyme. Our group constructed the modular peptide bioprobe FC-PyTPA by linking PyTPA with peptides [79]. FC-PyTPA (λ_ex_: 450 nm, λ_em_: 650 nm) consisted of three elements: an amphiphilic structure F with a 16-carbon alkyl chain and a peptide element (GGGH) which can self-assemble to form nanofibers and kill cancer cells; a positively charged penetrating peptide (GRKKRRQRRR) defined as a C element which could transport therapeutic genes (siRNA) into cells through electrostatic interactions; and an AIEgen (PyTPA) element which was used for imaging-guided photodynamic therapy. In the presence of matrix metalloproteinases-2 (MMP-2), FCsiRNA-PyTPA was specifically cleaved into two parts: FCsiRNA and PyTPA. FCsiRNA could be hydrolyzed by CB in lysosomes and promoted the formation of nanofibers through molecular self-assembly. The other part, PyTPA, could image tumor cells for a long time.

**Figure 6:**
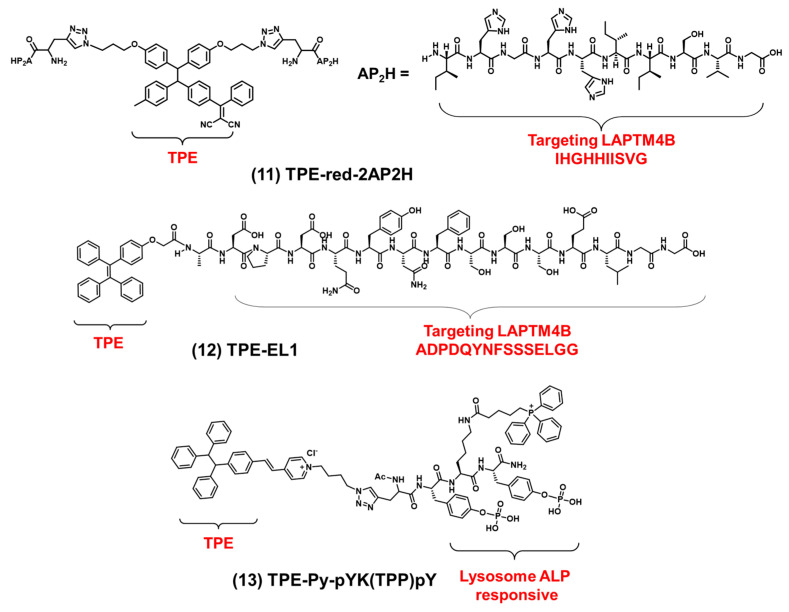
The chemical structures of lysosome-targeting probes.

Using lysosomal proteins as driving forces for targeting lysosomes. Zhang et al. synthesized a bioprobe, TPE-RED-2AP2H (λ_ex_: 445 nm, λ_em_: 620 nm) [80]. As shown in Figure 6 compound (**11**), the bioprobe consisted of TPE and two AP2H peptides (IHGHHIISVG). The AP2H peptide could target lysosomal protein transmembrane 4 beta (LAPTM4B). Using this property, TPE-RED-2AP2H successfully tracked the movement of the LAPTM4B protein from the cell membrane to the lysosomes in cells. In addition, the bioprobe could produce ROS under visible light irradiation, which can be used in photodynamic therapy. The bioprobe had the dual function of imaging proteins on lysosomes and photodynamic therapy. It was expected to be widely used in biological research. Based on protein-targeting strategies in lysosomes, Zhang et al. synthesized the bioprobe EL1-TPE (λ_ex_: 330 nm, λ_em_: 470 nm) by linking TPE with the EL1 peptide (ADPDQYNFSSSELGG) [81]. As shown in Figure 6 compound (**12**), the EL1 peptide also could target LAPTM4B. Co-localization experiments showed that EL1-TPE and LysoTracker overlapped very well. EL1-TPE showed excellent properties for lysosome tracking. In addition to targeting proteins in lysosomes, bioprobes can also respond to proteins in lysosomes, such as alkaline phosphatase (ALP) responding. As shown in Figure 6 compound (**13**), Ding et al. reported an AIEgen-peptide bioprobe (TPE-pY-pYK(TPP)pY) which can respond to ALP and then target lysosomes [82]. The bioprobe was composed of TPE, phosphorylated peptides and triphenylphosphine (TPP). The excitation wavelength of the bioprobe is 400 nm, and the maximum emission wavelength is 595 nm. The co-localization experiments showed that the bioprobe was mainly concentrated in lysosomes (Pearson correlation coefficient: 0.72) rather than mitochondria (Pearson correlation coefficient: 0.24). Although triphenylphosphine targeted mitochondria, the bioprobe accumulated primarily in lysosomes due to its surface charge and nanoscale size and the enzymatic response of phosphorylated peptides to ALP. As a lysosomal membrane permeability-inducer (LMP), TPE-pY-pYK(TPP)pY not only imaged lysosomes but also induced immunogenic cell death (ICD) and could efficiently convert immune cold tumors into hot tumors. This work established a new bridge between lysosome-associated cell imaging and cell death.

### 2.5. Endoplasmic Reticulum-Targeted Bioprobes

The endoplasmic reticulum is a tubular structure made up of a single membrane that forms a continuous omental system. It plays an important role in protein synthesis, modification, folding, transportation and assembling new peptide chains [9,10,11]. The endoplasmic reticulum is usually divided into the rough endoplasmic reticulum and the smooth endoplasmic reticulum according to whether there are ribosomes attached to the outer surface of the endoplasmic reticulum. Folded proteins are transported to the Golgi apparatus by the endoplasmic reticulum, while misfolded or unfolded proteins are transferred to lysosomes for degradation. When misfolded proteins were transported into the endoplasmic reticulum, it could cause endoplasmic reticulum stress, which led to a variety of diseases including diabetes, neurodegenerative diseases and inflammation. Therefore, it is of great significance to develop fluorescent probes for endoplasmic reticulum physiological state monitoring. KDEL peptides are often used as peptide sequences targeting the endoplasmic reticulum [83]. Benzenesulfonamide is a chemical structure commonly used to target the endoplasmic reticulum [84]. Cho et al. synthesized two endoplasmic reticulum-targeted two-photon fluorescent probes (BER-blue and FER-green) [85]. They linked fluorophores with the KDEL peptide to image the endoplasmic reticulum in cells and tissues. The KDEL peptide could localize to the endoplasmic reticulum within 5–15 min. These two fluorescent probes achieved the imaging of the endoplasmic reticulum efficiently and fast.

**Figure 7:**
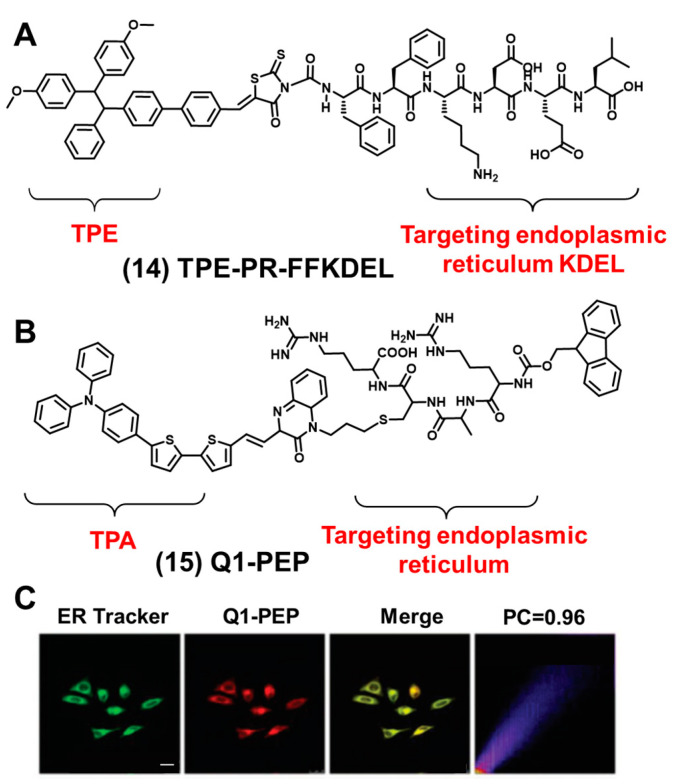
(**A**) Structural formula of TPE-PR-FFKDEL. (**B**) Structural formula of Q1-PEP. (**C**) Co-localization of Q1-PEP and the endoplasmic reticulum tracker (this figure was adapted from [87] with some modifications). The scale bar is 25 μm.

As illustrated in Figure 7A, Ding et al. synthesized an endoplasmic reticulum-targeting AIEgen-peptide bioprobe (TPE-PR-FFKDEL) consisting of AIEgen (TPE) and an endoplasmic reticulum-targeted peptide KDEL. The excitation wavelength of the bioprobe is 430 nm, and the maximum emission wavelength is 620 nm. A co-localization experiment showed that the Pearson correlation coefficient is 0.923 [86]. AIEgen showed unique fluorescence properties, and the peptide KDEL showed an excellent performance in the endoplasmic reticulum co-location experiment. After entering 4T1 cells, the bioprobe could anchor on the endoplasmic reticulum to produce a large number of ROS, induce the immunogenic cell death caused by endoplasmic reticulum oxidative stress effectively and promote tumor immunotherapy. The successful preparation of TPE-PR-FFKDEL not only improved the endoplasmic reticulum-targeting ability but also provided a novel effective photosensitizer. All these results demonstrated the significant advantages and prospects of AIEgen-peptide bioprobes in imaging the endoplasmic reticulum.

As we can see from Figure 7B, Zhang et al. reported a bioprobe Q1-PEP composed of an amphiphilic quinoxalinone derivative-peptide and AIEgen [87]. The excitation wavelength of the bioprobe is 480 nm, and the maximum emission wavelength is 653 nm. All of the results showed that Q1-PEP could enter the endoplasmic reticulum effectively and had excellent endoplasmic reticulum-targeting ability; the co-localization coefficient reached 0.96 (Figure 7C). The endoplasmic reticulum is mainly involved in protein synthesis in vesicles and transports to the Golgi apparatus or lysosomes. Q1-PEP could not only be used for endoplasmic reticulum imaging but also for detecting vesicle transport in living cells. Q1-PEP had a large Stokes shift and stable AIE properties. Given these unique properties, Q1-PEP could be further used to study endoplasmic reticulum interactions with other organelles and to track vesicle transports involving biological processes over time.

## 3. Conclusions and Future Prospects

In conclusion, this review summarized the application of AIEgen-peptide bioprobes in organelles imaging according to organelle types. The addition of peptides endowed AIEgens with better biocompatibility, diversified biological functions and organelles-targeting ability. According to the structural characteristics of organelles, we introduced the targeting mechanisms, targeting strategies and biological applications of AIEgen-peptide bioprobes. These organelles include the cell membrane, nucleus, endoplasmic reticulum, lysosomes and mitochondria. Despite achieving great progress in organelle imaging, AIEgen-peptide bioprobes targeting organelles still have a great potential for development, including but not limited to the following issues: (1) Most AIEgen-peptide bioprobes are designed for the cell membrane, but fewer are designed for other organelles such as the mitochondria, nucleus, lysosomes, Golgi apparatus and endoplasmic reticulum. The reason for this may be that the study of proteins in organelles is not clear. So, we still have a long way to go in this area. Most of these low-profile organelles-targeted bioprobes should be discovered in the future. (2) The peptide sequence targeting organelles is relatively singular. According to the structural characteristics of organelles, the future research direction will design more organelles-targeted peptides by modulating the peptide sequence. (3) Bioprobes’ entrance into cells is greatly affected by enzymes, and the stability of bioprobes could be improved by structural modification as far as possible. (4) Some bioprobes with a better optical performance need to be developed, such as near-infrared bioprobes and super-resolution bioprobes. Although AIEgen-peptide bioprobes have some difficulties in organelle imaging, we believe that these difficulties will be resolved and that they will become effective detection and treatment methods in the biomedical field.

**Table 1:** Summary of AIEgen-peptide bi oprobes.

Target	Peptide	AIEgen	Responsive Site	λ_ex_/λ_em_ (nm)	Cytotoxicity	References
Cell membrane	cRGD	TPS	α_v_β_3_	356/480	no cytotoxicity	[53]
	RRRR	TPE		330/466	no cytotoxicity	[54]
	RGD-Pal-RRRR	T-MY		330/500	no cytotoxicity	[55]
	C_16_-K(TPE)-GGGH-GFLGK-PEG_8_	TPE		370/470	IC_50_ = 0.1 mg/mL	[56]
	YCDGFYACYMDV	BP	HER2	380/520	no cytotoxicity	[57]
	YSAYPDSVPMMS	DBT	EphA2	490/642	IC_50_ = 38.3 × 10^−6^ M	[58]
Nucleus	NLS	PyTPE		405/570	no cytotoxicity	[65]
	NLS	PyTPE		405/580	no cytotoxicity	[66]
Mitochondria	HLAHLAHHLAHLAH	TPE		420/720	no cytotoxicity	[76]
	klaklakklaklak	PyTPA		450/620	High cytotoxicity	[77]
Lysosome	GFLG	PyTPA	CB	450/650	High cytotoxicity	[79]
	IHGHHIISVG	TPE	LAPTM4B	445/620	EC_50_ = 3.1 μM	[80]
	ADPDQYNFSSSELGG	TPE	LAPTM4B	330/470	no cytotoxicity	[81]
	pYK(TPP)pY	TPE	ALP	400/595	IC_50_ = 9.7 μM	[82]
Endoplasmic reticulum	KDEL	TPE		430/620	no cytotoxicity	[86]
		TPA		480/653	no cytotoxicity	[87]

Abbreviations: NLS, nuclear localization signal; CB, cathepsin B; LAPTM4B, lysosomal protein transmembrane 4 beta; ALP, alkaline phosphatase.

## Data Availability

Not applicable.
